# Development of a novel prognostic model based on TRPM4-Induced sodium overload–mediated cell death in kidney cancer

**DOI:** 10.3389/fcell.2025.1755318

**Published:** 2026-01-09

**Authors:** Wei Wang, Duo Zhao, Zijun Zhou, Bin Chen, Changwen Zhang, E. Du, Longchao Zhang

**Affiliations:** Department of Urology, Tianjin Institute of Urology, The Second Hospital of Tianjin Medical University, Tianjin, China

**Keywords:** bioinformatics, cell death, clear cell renal cell carcinoma (ccRCC), prognostic model, TRPM4, tumor immune microenvironment

## Abstract

**Background:**

Clear cell renal cell carcinoma (ccRCC) is the predominant subtype of kidney cancer. Its incidence and mortality rates remain consistently high, creating an urgent need to identify novel biomarkers and therapeutic targets. Necrosis by sodium overload (NECSO), mediated by the TRPM4 channel, represents a newly discovered form of cell death; however, its role in ccRCC remains unclear.

**Methods:**

We performed a pan-cancer analysis of TRPM4 using TCGA data. GO, and KEGG enrichment analyses were employed to investigate TRPM4-associated functions and pathways in KIRC. Three machine learning algorithms (plsRcox, GBM, and CoxBoost) were integrated to identify 14 pivotal genes for constructing a comprehensive NECSO Score. TIME was assessed using CIBERSORT, xCell, and ESTIMATE algorithms. Finally, the biological functions of TRPM4 were validated in 769-P and A498 cells through *in vitro* experiments.

**Results:**

Pan-cancer analysis revealed that TRPM4 was significantly downregulated in KIRC, and its high expression was associated with prolonged RFS. The NECSO Score, derived from the 14-gene signature, served as an independent protective prognostic factor. A high NECSO Score was correlated with an activated immune microenvironment, characterized by increased infiltration of CD8^+^ T cells and Th1 cells. *In vitro* assays confirmed that TRPM4 overexpression suppressed the proliferation, migration, and clonogenicity of ccRCC cells while promoting apoptosis. Furthermore, TRPM4 overexpression synergized with the sodium overload inducer Necrocide-1 (NC1) to enhance anti-tumor efficacy.

**Conclusion:**

This study systematically unveils the tumor-suppressive role of TRPM4 in ccRCC and innovatively establishes the NECSO Score as a robust prognostic model. This score not only accurately predicts patient outcomes but also illuminates the potential link between sodium ion homeostasis and the tumor immune landscape. Targeting TRPM4 and NECSO may represent a promising therapeutic avenue for ccRCC.

## 1 Introduction

In adult populations, the majority of kidney malignancies are diagnosed as renal cell carcinoma (RCC), which accounts for nearly 80%–85% of cases (Petejova and Martinek, 2016). In recent years, the incidence of RCC has shown a significant increase globally (Ljungberg et al., 2015; Feng et al., 2025). For example, GLOBOCAN 2020 estimated about 431,288 new cases of kidney cancer worldwide (Sung et al., 2021; Li et al., 2023). Renal cell carcinoma, therefore, accounts for roughly 2%–3% of all cancers globally, and incidence has been rising in recent years at an estimated annual rate of approximately 2% (Bukavina et al., 2022; van den Brink et al., 2024). Recent epidemiologic assessments estimate that RCC accounts for approximately 295,000 new cases and 134,000 deaths each year worldwide (Wang et al., 2017). Among its histologic subtypes, clear cell renal cell carcinoma (ccRCC) is the predominant form, representing more than 75% of all diagnoses (Doppalapudi et al., 2021). Despite advances in systemic therapy, the prognosis for patients with metastatic ccRCC remains poor, with a 5-year survival rate typically falling below 10% (Turajlic et al., 2018). Early diagnosis of RCC is highly challenging, with more than 60% of cases detected incidentally with routine imaging (van den Brink et al., 2024; Hoffler et al., 2015). Recurrent loss of the short arm of chromosome 3 (3p) is a defining genomic abnormality in ccRCC. This early event contributes to the two-hit inactivation of key tumor-suppressive loci, particularly VHL, thereby initiating the molecular cascade that drives ccRCC development (Hu et al., 2024). Clinical manifestations including hematuria, low back pain, and abdominal mass usually appear at advanced stages. In terms of molecular mechanisms, the heterogeneity of RCC is closely associated with mutations in PBRM1, SETD2 and BAP1 (Turajlic et al., 2018; Kumar et al., 2018). Among them, mutation or methylation of the VHL gene (seen in 90% of sporadic ccRCC) promotes tumor progression through aberrant accumulation of hypoxia-inducible factor (HIF), which activates angiogenic and glycolytic pathways (Kumar et al., 2018; Bahadoram et al., 2022). In addition, other subtypes such as papillary renal cell carcinoma (15% of cases) and chromophobe renal cell carcinoma (5% of cases) were associated with MET gene mutations and dysregulation of the TP53/PTEN pathway, respectively (Cancer Genome Atlas Research et al., 2016; Hsieh et al., 2018). Notably, the evolutionary trajectory of RCC is complex and diverse, encompassing linear, branching, or mutation burst patterns, which are closely linked to the tumor’s metastatic potential and resistance to treatment (Turajlic et al., 2018; Kowalewski et al., 2020). Although therapies targeting VEGF signaling and immune-checkpoint pathways have substantially improved outcomes for patients with advanced RCC, their long-term clinical efficacy remains limited. Both intrinsic and acquired resistance frequently emerge during treatment, and considerable variability in patient prognosis persists (Jonasch et al., 2014). Therefore, in-depth exploration of novel cell death pathways in RCC may provide critical insights to overcome current therapeutic bottlenecks.

As a central regulator of intra- and extracellular ionic homeostasis, an imbalance in sodium ion (Na^+^) homeostasis is closely associated with various pathological processes (Monteil et al., 2024). Excessive sodium inward flow can lead to collapse of the cell membrane potential and osmotic swelling, which in turn triggers a unique form of necrotic cell death, known as sodium overload-induced cell death (Shi et al., 2025). Unlike conventional apoptosis, sodium death is not dependent on caspase activation but is directly triggered by disruption of the transmembrane sodium gradient. It has been demonstrated that the transient receptor potential channel TRPM4 plays a crucial role in this process. As a calcium-activated, non-selective cation channel, the aberrant opening of TRPM4 amplifies sodium inward flow, resulting in a surge in intracellular osmotic pressure and membrane rupture. Notably, sodium death differs significantly from programmed necrosis, such as iron death (ferroptosis) and necroptosis, in that its specificity relies on sodium overload rather than iron accumulation or RIPK signaling pathways (Mansour et al., 2023). For example, in the necroptosis apoptosis model, sodium inward flow was shown to be a key downstream event following MLKL complex activation, whereas no similar phenomenon was observed during apoptosis (Liu et al., 2024).

In recent years, the discovery of the small molecule compound Necrocide-1 (NC1) has provided a new direction for targeting sodium death (Zhang et al., 2023). NC1 selectively induces necrosis in cancer cells at nanomolar concentrations without significant toxicity to normal cells. The mechanism involves sustained activation of TRPM4 channels, leading to uncontrolled sodium inward flow, membrane depolarization, and mitochondrial dysfunction (Xue et al., 2023). Notably, NC1-induced death was accompanied by immunogenic features, suggesting a potential antitumor immune-activating effect (Zhang et al., 2023). This property distinguishes it from conventional chemotherapeutic agents and provides a dual advantage for cancer treatment: direct killing of tumor cells and an enhanced immune response. Although the mechanisms of sodium death in a variety of diseases are becoming clearer, its role in tumor biology remains to be explored in depth. Metabolic abnormalities characterizing tumor cells may exacerbate cellular stress by affecting sodium gradients (Soll et al., 2024). For example, inhibition of sodium-potassium pump activity in the tumor microenvironment can lead to intracellular sodium accumulation, which in turn triggers calcium overload via the sodium-calcium exchanger reverse mode. This process is directly associated with cell death in ischemia-reperfusion injury (Bejcek et al., 2021). However, there is still a gap in research on how sodium homeostasis imbalance affects tumor progression and response to therapy.

In this study, we comprehensively characterized the expression profile of TRPM4 across multiple tumor types through systematic bioinformatics analysis, with a particular focus on evaluating its clinical relevance and prognostic value in clear cell renal cell carcinoma (KIRC). By integrating Gene Ontology (GO) and KEGG pathway enrichment analyses, we preliminarily inferred the biological functions and signaling pathways potentially involving TRPM4 and its associated genes. Concurrently, we constructed a necrosis by sodium overload (NECSO) scoring model based on TRPM4-associated gene features to predict patient survival outcomes. Furthermore, utilizing algorithms such as CIBERSORT and xCell, we systematically analyzed the relationship between NECSO scores, TRPM4 expression levels, and tumor immune cell infiltration characteristics. *In vitro* functional experiments further supported the tumor-suppressive role of TRPM4 in renal carcinoma and its negative correlation with adverse tumor phenotypes. Collectively, these findings demonstrate the potential clinical utility of TRPM4 and the NECSO scoring model for risk stratification and prognostic assessment in KIRC.

## 2 Materials and methods

### 2.1 Pan-cancer data Collection and processing

We obtained RNA-sequencing data for 33 tumor types from the TCGA database via the UCSC Xena platform (https://xena.ucsc.edu/). This pan-cancer cohort encompassed a broad spectrum of malignancies.

To characterize TRPM4 expression patterns across human cancers, transcriptomic data from the TCGA cohort were analyzed. TRPM4 mRNA levels were assessed in tumor samples and, when available, corresponding normal tissues across 33 cancer types. Expression differences were examined within the R environment, and visualization of TRPM4 variation among cancer types, subgroups, or pathological stages was generated using box-plot-based graphics implemented with the ggplot2 package.

### 2.2 Differential expression analysis of TRPM4

Differential expression was assessed using the limma package, and gene-level comparisons were conducted with the Wilcoxon signed-rank test and the Benjamini–Hochberg correction. Transcripts with an adjusted P < 0.05 and |log_2_FC| > 0.75 were defined as significant. Statistical annotations followed the convention: *P* < 0.05; *P < 0.01; **P < 0.001; ***P < 0.0001; NS, not significant.

### 2.3 Survival analysis

Cox proportional hazards models and Kaplan-Meier survival curves were used to evaluate the prognostic relevance of TRPM4 expression, with analyses performed using the’survival’and’survminer’packages in R. The hazard ratio (HR) associated with TRPM4 levels was first estimated using a univariate Cox model. Subsequently, multivariate Cox regression was conducted to obtain the adjusted HR after controlling for clinical variables.

### 2.4 GO, KEGG analyses, and PPI

Functional enrichment of TRPM4-related genes was performed using clusterProfiler for Gene Ontology and KEGG analyses. The TRPM4 protein-protein interaction network was constructed through GeneMANIA (https://genemania.org/).

### 2.5 Construction and validation of the predictive model

Univariate Cox regression was used to screen DEGs and clinical variables associated with recurrence-free survival, and factors with *P* < 0.05 were entered into multivariate Cox analysis to identify independent predictors. Model selection was optimized using stepwise regression based on the minimum AIC. A prognostic nomogram was subsequently constructed in the rms package by integrating the selected variables and risk scores. Model performance was assessed using the concordance index, time-dependent ROC curves, and calibration plots. Clinical utility was evaluated through decision curve analysis.

### 2.6 Immune cell infiltration analysis

To comprehensively characterize immune cell infiltration and the tumor immune microenvironment (TIME), we employed four computational algorithms: CIBERSORT, ssGSEA, xCell, and ESTIMATE. The xCell R package was used to quantify the infiltration levels of various immune cells and to calculate the immune score, stromal score, and microenvironment score for each sample. CIBERSORT was applied to estimate the relative proportions of 22 immune cell types per sample based on gene expression profiles. The ssGSEA algorithm was used further to assess immune-related pathway enrichment at the individual sample level. ESTIMATE was utilized to infer tumor purity and the relative presence of stromal and immune components.

Comparisons of immune cell infiltration and immune scores between groups were conducted using the Wilcoxon rank-sum test. Statistical significance was defined as *P* < 0.05. All visualizations were generated using the “ggplot2” package in R (version 4.4.3).

### 2.7 Cell culture and reagents

We conducted experiments using human renal carcinoma cell lines 769-P and A498. Both cell lines were cultured in medium containing 10% fetal bovine serum (FBS; HAKATA, United States) and 1% penicillin/streptomycin (Hyclone, United States) at 37 °C with 5% CO_2_ humidity. 769-P cells were cultured in RPMI-1640 medium (Gibco, United States), while A498 cells were cultured in Dulbecco’s Modified Eagle Medium (DMEM; Gibco, United States). TRPM4 overexpression was achieved via plasmid transfection, with the overexpressed TRPM4 designated as TRPM4-OE and the negative control referred to as Vector.

### 2.8 Western blot

We proceeded using the established protocol (Zhang et al., 2025). Protein concentrations were measured using the BCA method, followed by electrophoresis separation and transfer to PVDF membranes. After blocking, primary antibodies against TRPM4 and GAPDH were incubated overnight at 4 °C. Secondary antibodies labeled with HRP were incubated at room temperature for 1 h. Protein signals were detected using enhanced chemiluminescence, and band intensities were quantified using ImageJ software.

### 2.9 Clone formation assay

Inoculate the processed cells into six-well plates or 12-well plates containing DMEM or RPMI 1640 medium supplemented with 10% fetal bovine serum, and culture them typically for 2 weeks. Subsequently, fix the cells with 4% paraformaldehyde, stain with a 1% crystal violet solution, and finally count the number of cell colonies using ImageJ software.

### 2.10 Flow cytometry

To assess apoptosis after overexpression of TRPM4 in both cells, we used the Annexin V-FITC/Propidium Iodide Apoptosis Detection Kit according to the manufacturer’s instructions. Apoptotic cell populations were quantified using the FACScan flow cytometry system (BD Biosciences). Data were analyzed using FlowJo software, and the corresponding cells were detected by flow cytometry.

### 2.11 Statistical analysis

All analyses were performed using R version 4.4.3. Group differences were evaluated using one-way ANOVA or Student’s t-test as appropriate. Survival was assessed via Kaplan-Meier analysis with log-rank tests or Cox proportional hazards models. Pearson or Spearman correlation coefficients were used to examine the associations, with |r| ≥ 0.3 considered statistically significant.

## 3 Results

### 3.1 Pan-cancer analysis of TRPM4 expression

**FIGURE 1:**
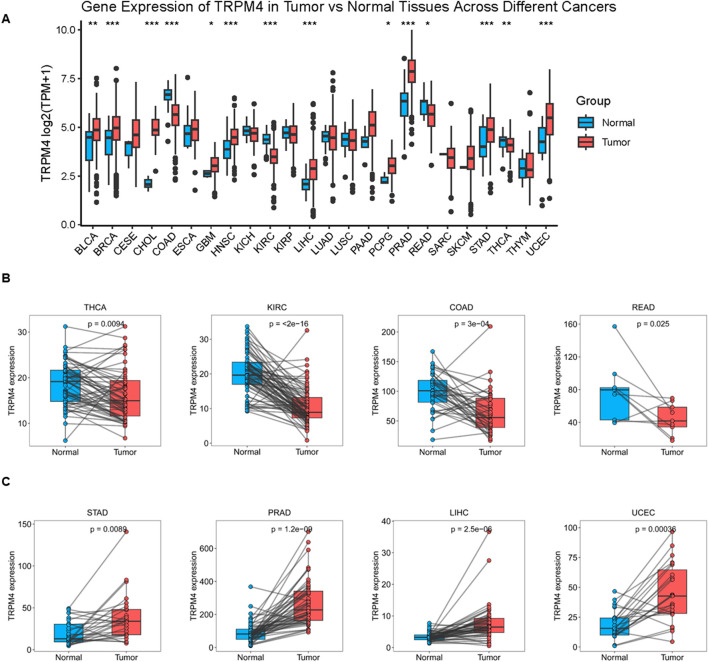
Expression of TRPM4 mRNA in pan-cancer **(A)** Comparison of TRPM4 expression between tumor and standard samples in 33 cancer types obtained from the TCGA database (*P < 0.05; **P < 0.01; ***P < 0.001; ns, not significant); **(B)** Comparison of TRPM4 expression between tumor and paired standard samples in THCA, KIRC, COAD, READ from the TCGA database (*P < 0.05; **P < 0.01; ***P < 0.001; ns, not significant). **(C)** Comparison of TRPM4 expression between tumor and paired standard samples in STAD, PRAD, LIHC, UCEC from the TCGA database (*P < 0.05; **P < 0.01; ***P < 0.001; ns, not significant).

To explore the potential relevance of TRPM4 in cancer, we initially surveyed its mRNA expression across 33 different tumor types utilizing data from TCGA. The results show that TRPM4 was downregulated in COAD, KIRC, READ and THCA. However, it was upregulated in LIHC, BRCA, CHOL, GBM, BLCA, HNSC, PCPG, UCEC, STAD and PRAD (Figure 1A). Furthermore, the paired comparative analysis showed that TRPM4 expression was significantly lower in COAD, KIRC, THCA, and READ compared to adjacent normal tissues (Figure 1B). In contrast, higher TRPM4 expression was observed in tumor tissues of STAD, PRAD, LIHC, UCEC, PCPG, PAAD, CHOL and HNSC (Figure 1C, Supplementary Figure S1).

**FIGURE 2:**
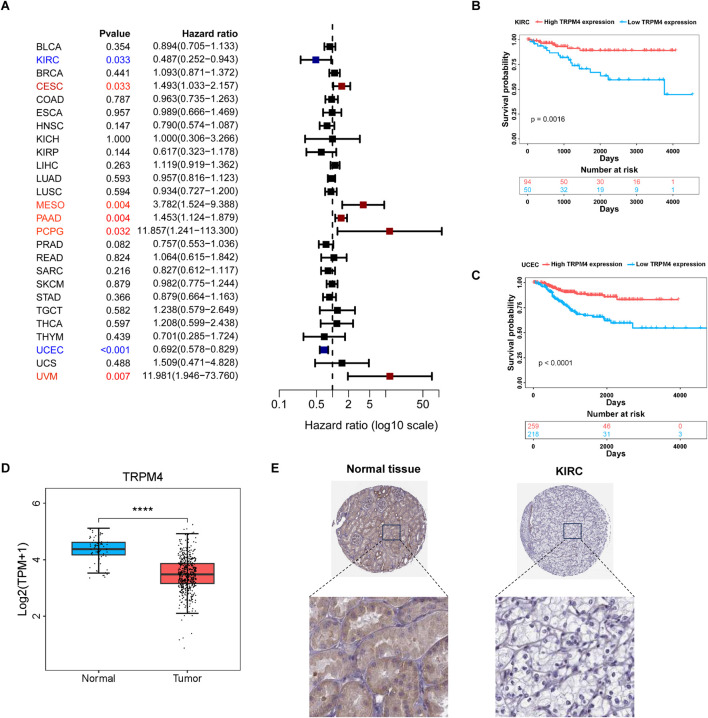
Prognostic analysis of TRPM4 in different cancer types **(A)** Forest plot showing the correlation between TRPM4 expression and RFS in various cancers; **(B,C)** Kaplan-Meier analysis showing that higher TRPM4 expression is associated with improved RFS in KIRC and UCEC patients. **(D)** expression of TRPM4 in KIRC in TCGA; **(E)** Representative immunohistochemical staining images of TRPM4 in normal kidney tissue and renal cell carcinoma tissue.

To investigate the prognostic significance of TRPM4 expression in 33 different types of cancer, we performed survival analysis using Cox proportional hazards modeling and assessed the correlation between TRPM4 expression and recurrence-free survival (RFS). Univariate Cox analysis indicated that higher TRPM4 expression was associated with improved RFS in both KIRC and UCEC (Figure 2A). This result was also validated by Kaplan-Meier curves showing that elevated TRPM4 expression was significantly associated with prolonged RFS survival in KIRC, STAD and HNSC (Figures 2B,C, Supplementary Figure S2). Conversely, patients with high TRPM4 expression levels had a worse prognosis in PCPG (Supplementary Figure S2). We found that TRPM4 expression in tumors was lower than in the normal group in the KIRC model (Figure 2D). This was subsequently validated through immunohistochemical analysis, which confirmed the reduced TRPM4 expression levels in tumor tissues (Figure 2E). These results suggest that TRPM4 is downregulated in KIRC, and elevated TRPM4 expression is significantly associated with improved patient prognosis. Thus, TRPM4 is not only implicated in KIRC pathogenesis but also represents a potential prognostic biomarker for this disease.

### 3.2 Screening for TRPM4-related modules and genes in KIRC

**FIGURE 3:**
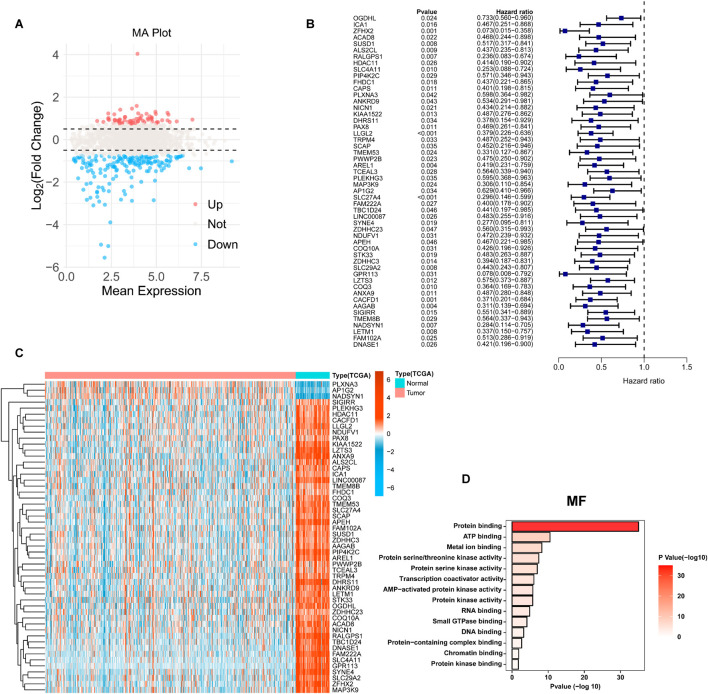
Identification of differentially expressed genes (DEGs). **(A)** The MA plot displays the differentially expressed genes between KIRC and controls in the TCGA-KIRC datasets. **(B)** The forest diagram shows 52 genes. **(C)** Heatmaps show the expression levels of 52 genes in tumors and normal tissues. **(D)** GO analysis for DEGs in MF term.

To systematically identify molecules significantly correlated with TRPM4 expression, we calculated the Pearson correlation coefficients (PCCs) between TRPM4 and genome-wide gene expression profiles using transcriptomic data from TCGA-KIRC tumor samples. Genes positively correlated with TRPM4 (PCC >0.3) were selected as candidate targets. Differential expression analysis was then performed between tumor and normal tissues, with filtering criteria set as |log2FoldChange| > 0.75 and adjusted *P*-value <0.05. A total of 1,664 differentially expressed genes (DEGs) were identified (Figure 3A). These DEGs were integrated with clinical follow-up data, and their associations with RFS in KIRC were assessed using univariate Cox regression, with significance defined as *P* < 0.05. Ultimately, 52 genes were identified whose expression levels were significantly associated with patient RFS. Notably, forest plot analysis revealed that all 52 genes had hazard ratios (HRs) less than 1, suggesting that these genes exhibit a protective expression pattern in KIRC and may serve as potential prognostic biomarkers (Figure 3B). This result was further validated by the heatmap results, which showed that the vast majority of genes were upregulated in normal tissues and significantly downregulated in tumor tissues (Figure 3C). To further investigate the potential biological functions and pathways of TRPM4 and 51 other related conserved genes, we performed Gene Ontology (GO) enrichment and Kyoto Encyclopedia of Genes and Genomes (KEGG) pathway analysis. We constructed a protein-protein interaction (PPI) network (Supplementary Figure S3D). GO enrichment analysis revealed that, at the Molecular Function (MF) level (Figure 3D), the genes were significantly enriched in terms such as protein binding, ATP binding, metal ion binding, and kinase activity. In the Biological Process (BP) category, these genes were mainly involved in chromatin remodeling, protein transport, autophagy, TORC1 signaling, and vesicle-mediated transport. In the Cellular Component (CC) domain, the enriched terms included cytosol, nucleoplasm, mitochondria, and endosomal membrane (Supplementary Figure S3A,B). KEGG pathway analysis (Supplementary Figure S3C) showed significant enrichment in lysosome, autophagy, mTOR signaling pathway, and metabolic pathways.

### 3.3 Integrative machine learning-based development and validation of diagnostic signatures

**FIGURE 4:**
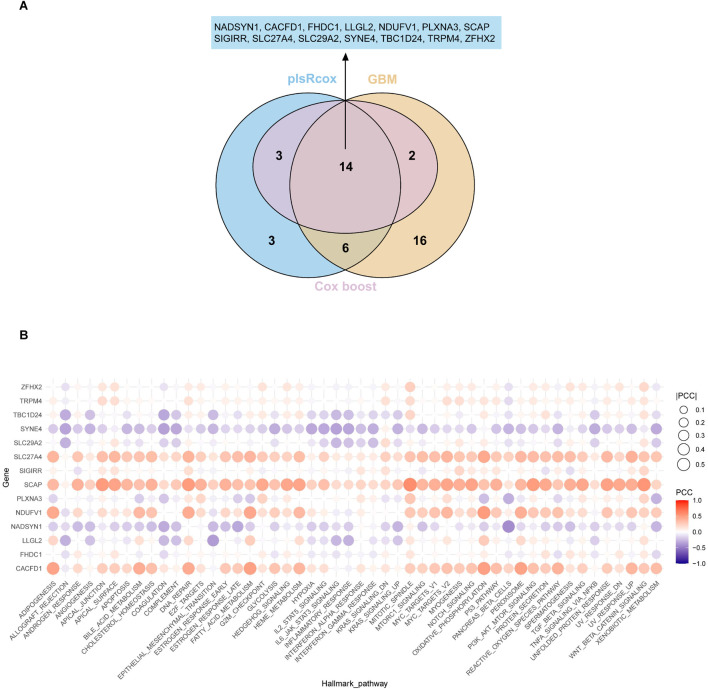
Construction of the NECSO Score using machine-learning algorithms. **(A)** A Venn diagram illustrates the machine learning process. **(B)** Correlation landscape between the 14-gene diagnostic signature and Hallmark pathways in KIRC.

To construct diagnostic features based on the KIRC, we employed three machine learning algorithms, plsRcox, Gradient Boosting Machine (GBM), and CoxBoost for variable selection and further model refinement. Ultimately, we identified 14 key genes: NADSYN1, CACFD1, FHDC1, LLGL2, NDUFV1, PLXNA3, SCAP, SIGIRR, SLC27A4, SLC29A2, YNE4, TBC1D24, TRPM4, and ZFHX2 (Figure 4A). The survival analyses of these genes are presented in Supplementary Figure S4. Subsequently, we performed Hallmark gene set enrichment analysis to explore their functional distribution in critical biological processes (Figure 4B). Notably, TRPM4 emerged as a pivotal gene involved in several essential cellular processes, showing significant enrichment in pathways such as hypoxia, inflammatory response, apoptosis, and oxidative phosphorylation. These findings suggest that TRPM4 may play a crucial role in regulating cellular stress responses, cell death, and metabolic homeostasis. Although traditional genome enrichment analysis (GSEA) and pathway annotation methods have provided insights into the mechanisms underlying necrosis by sodium overload (NECSO), they remain limited in capturing the pathway specificity and biological nuances of this process. Most of the existing enrichment methods rely heavily on predefined pathway databases, which may overlook key signaling axes uniquely activated during sodium overload-induced cell death. To address this limitation, we incorporated the 14 diagnostic signatures as the defining gene set to derive an integrated scoring model within the NECSO framework, hereafter termed the NECSO Score. The formula is as follows: NECSO Score = ssGSEAscore (14 diagnostic signatures).

**FIGURE 5:**
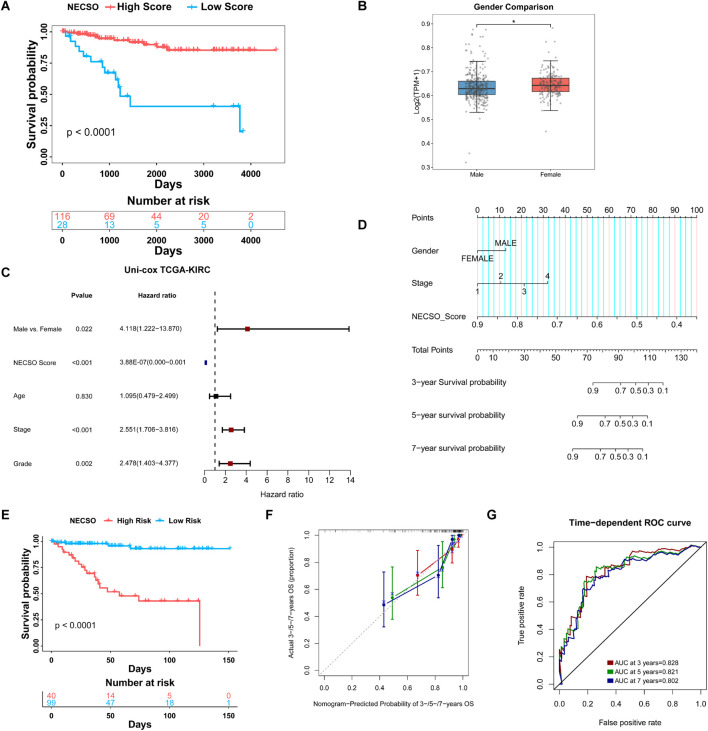
Prognostic and clinicopathological associations of the NECSO Score in TCGA-KIRC cohort. **(A)** Kaplan-Meier survival analysis revealed that patients in the High NECSO Score group had significantly better recurrence-free survival (RFS) than those in the Low Score group. **(B)** Distribution of NECSO Scores in the Gender Subgroup. **(C)** Univariate Cox proportional hazards analysis showing hazard ratios (HRs) for RFS according to NECSO Score and clinical parameters. **(D)** Nomogram predicting 3-, 5-, and 7-year survival probabilities for patients in the KIRC cohort. **(E)** Kaplan-Meier curves comparing survival between nomogram-defined High- and Low-risk groups. **(F)** Calibration plots showing the consistency between nomogram-predicted and actual survival probabilities at 3, 5, and 7 years. **(G)** Time-dependent ROC analysis of the combined model.

To further assess the prognostic significance of the NECSO Score and its relationship with clinical features, we divided patients in the TCGA-KIRC cohort into High-Score and Low-Score groups according to the optimal cutoff and performed a comprehensive evaluation. Kaplan-Meier analysis demonstrated significantly longer recurrence-free survival in patients with high NECSO scores compared to those with low scores (*P* < 0.0001) (Figure 5A). This finding was further validated in the independent external validation cohorts E-MTAB-1980 and TCGA-KIRP (Supplementary Figure S6A, S7A), both showing consistent survival stratification trends, indicating that the NECSO score possesses robust prognostic stratification capability and cross-cohort stability. Subsequently, we performed a comparative analysis of the correlation between the NECSO Score and multiple clinical parameters, including gender, age, pathological stage, and histological grade. Boxplot comparisons revealed a significant gender-related variation in the NECSO Score, with male patients displaying notably lower values than their female counterparts (*P* < 0.05) (Figure 5B). In contrast, the NECSO Score showed no meaningful variation across different age categories, pathological stages, or histological grades (*P* > 0.05) (Supplementary Figure S5A). Further Cox regression analysis showed that, in the univariate model, the NECSO Score was a significant protective factor for overall survival. At the same time, sex, pathological stage, and histological grade were also significantly associated with survival prognosis (Figure 5C). In the multivariate model incorporating multiple variables, the NECSO Score remained an independent and significant protective factor, whereas pathological stage emerged as an independent risk factor. Sex, age, and histological grade were not retained as substantial covariates in the multivariate analysis (Supplementary Figure S5B).

To facilitate individualized risk estimation in clinical settings, we constructed a nomogram incorporating the NECSO Score within a Cox proportional hazards model. This nomogram incorporates the NECSO Score alongside stage and gender to predict the 3-year, 5-year, and 7-year survival probabilities of KIRC patients. The nomogram results (Figure 5D) demonstrated that the contribution of the NECSO Score was negatively correlated with predicted survival probability, indicating its independent role as a protective factor within the model. Survival analysis revealed a significant divergence in outcomes between high- and low-risk patients, as classified by the integrated prognostic model (Figure 5E). To evaluate the nomogram, we assessed its predictive performance using time-dependent receiver operating characteristic (ROC) curves (Figure 5F). These curves showed that the combined model (incorporating the NECSO Score, Stage, and Gender) achieved high discrimination, with area under the curve (AUC) values of 0.828, 0.821, and 0.802 for 3-year, 5-year, and 7-year predictions, respectively (Figure 5G). Furthermore, the nomogram demonstrated good calibration across all three time points (3, 5, and 7 years), with the predicted survival probabilities showing excellent agreement with the actual observed survival rates. In addition, Decision Curve Analysis (DCA) was applied to assess the net clinical benefit of the NECSO Score, the individual clinical factors (Stage and Gender), and the integrated model (Supplementary Figure S5C-S5E). The DCA results indicated that both the NECSO Score alone and the combined model yielded substantial net clinical benefit for predictions at 3, 5, and 7 years. Crucially, the clinical net benefit of these models was superior to that offered by models based solely on a single clinical variable. Subsequently, we validated the NECSO scoring model in the independent external validation cohorts E-MTAB-1980 and TCGA-KIRP. Results demonstrated that the NECSO score consistently distinguished patient groups with different prognostic risks across these external datasets, exhibiting robust predictive performance (Supplementary Figure S6B-S6E, S7B-S7E). These findings further support the robustness and generalizability of the NECSO scoring model across independent cohorts.

### 3.4 Evaluation of TIME and immune checkpoint targets

**FIGURE 6:**
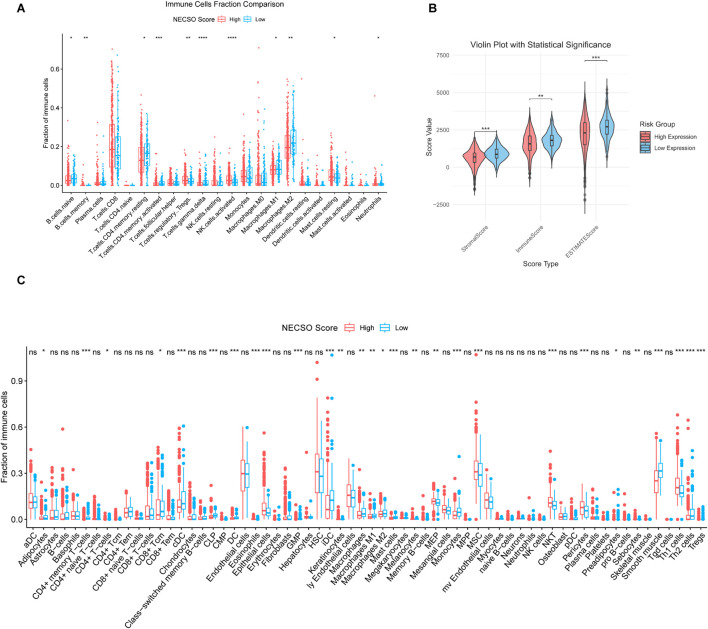
NECSO Score is correlated with immune-infiltrated cell populations in KIRC. **(A)** Proportions of immune-related cell types between the high- and low-score groups. **(B)** Comparison of ESTIMATE scores between NECSO high- and low-score groups. **(C)** Activity scores of immune-related cell types in the High- and Low-score groups.

We subsequently investigated the relationship between the TIME and the NECSO risk score. As shown in Figure 6A, the High-score group displayed substantial infiltration of immune-activating cells (including activated CD4^+^ T cells, CD8^+^T cells and NK cells). In contrast, immunosuppressive populations such as M2 macrophages were less abundant. Furthermore, the Estimate algorithm was used to calculate total Estimate scores, stromal scores, and immune scores across different NECSO score subgroups. The analysis revealed that the high-score group exhibited markedly reduced stromal and ESTIMATE scores (Figure 6B), suggesting that a higher NECSO Score may be associated with diminished stromal or immune infiltration and potentially increased tumor purity. In addition, xCell analysis revealed that NECSO High-score tumors exhibited significant enrichment of multiple immune cell types, including NKT cells and TH1 cells, while displaying a trend of downregulation in M2 macrophages and Treg cells (Figure 6C). These findings further support the notion that the NECSO Score is closely associated with a distinct and complex immune-stromal microenvironment in KIRC.

### 3.5 The expression validation of TRPM4 in KIRC

**FIGURE 7:**
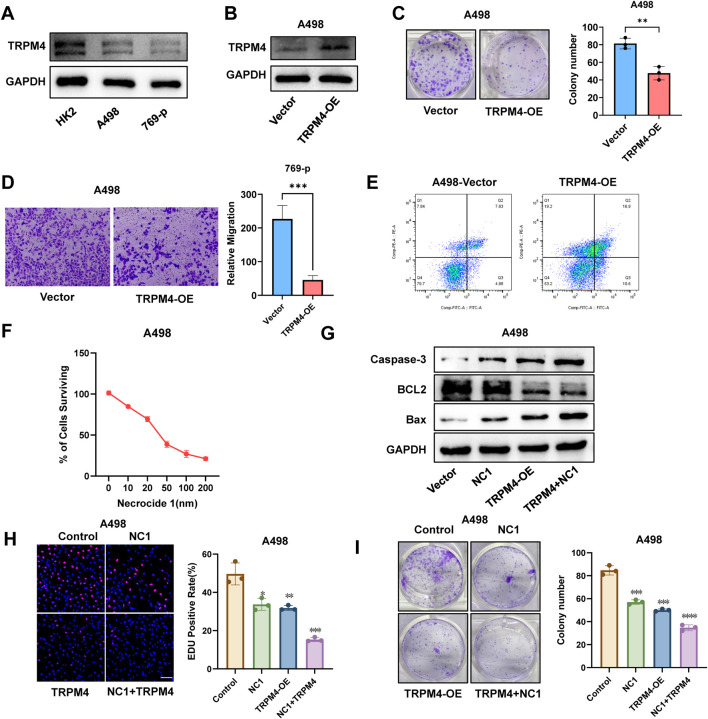
Overexpression of TRPM4 inhibits RCC progression while promoting programmed cell death in A498 cells. **(A)** Expression of TRPM4 in Normal Cells and RCC Cells. **(B)** Detection of TRPM4 expression in A498 cells by Western blot analysis. **(C)** Colony formation comparison between TRPM4-OE and control cells. **(D)** Transwell assays were used to evaluate the migration of TRPM4-OE cells compared to controls. **(E)** Flow cytometry analysis of apoptosis levels in control and TRPM4-OE cells. **(F)** Percentage of viable A498 cells following exposure to varying concentrations of NC1. **(G)** Detection of Caspase-3, Bax, and BCL-2 expression in A498 cells by Western blot analysis. **(H)** EdU assay showing that TRPM4 overexpression and the NC1 inhibitor suppress A498 cell proliferation. Scale bar = 200 µm. **(I)** Colony formation comparison between control cells, NC1, TRPM4-OE, and NC1+TRPM4-OE.

To investigate the role of TRPM4 in RCC, we first assessed TRPM4 expression in regular ccRCC cell lines (769-P and A498). Western blot analysis revealed a markedly reduced expression of TRPM4 in both cancer cell lines compared to normal controls (Figure 7A). To elucidate its functional role, we established TRPM4-overexpressing cell models and validated TRPM4 expression in A498 and 769-P cells (Figure 7B, Supplementary Figure S8A). TRPM4 overexpression significantly inhibited colony formation (Figure 7C, Supplementary Figure S8B) and reduced migratory capacity in both cell lines, as shown by Transwell assays (Figure 7D, Supplementary Figure S8C). Flow cytometry using Annexin V-FITC/PI staining revealed a substantial increase in both early and late apoptotic cells upon TRPM4 overexpression (Figure 7E, Supplementary Figure S8D), suggesting a pro-apoptotic, anti-tumor role for TRPM4. To further investigate whether TRPM4 exerts this effect through necrosis by sodium overload (NECSO), we introduced the necroptosis inhibitor Necrocide-1 (NC1). CCK-8 assays demonstrated a dose-dependent suppression of cell viability by NC1 in 769-P and A498 cells (Figure 7F, Supplementary Figure S8E). Western blot analysis confirmed that TRPM4 overexpression promoted a pro-apoptotic profile, consistent with increased levels of Bax and cleaved Caspase-3 alongside decreased BCL-2 expression. These effects were further enhanced upon combination with NC1 (Figure 7G, Supplementary Figure S8F), indicating a potential mechanistic link between TRPM4-mediated apoptosis and sodium overload–related cell death. EdU incorporation assays showed that both TRPM4 overexpression and NC1 monotherapy inhibited cell proliferation, while combination treatment had the most potent inhibitory effect (Figure 7H, Supplementary Figure S8G). Consistently, clonogenic assays validated the enhanced suppression of tumor cell growth by combined treatment (Figure 7I, Supplementary Figure S8H). These findings collectively indicate that TRPM4 overexpression suppresses the proliferative and migratory capacities of renal cancer cells while simultaneously promoting sodium overload-induced cell death.

## 4 Discussion

Clear cell renal cell carcinoma (ccRCC) remains a major therapeutic challenge due to its profound metabolic reprogramming (Zhu et al., 2023), substantial intratumoral heterogeneity (Hu et al., 2024), and intrinsic resistance to multiple forms of cell death (Zheng et al., 2025; Zhan et al., 2022). An increasing body of evidence suggests that both the initiation and progression of clear cell renal cell carcinoma (ccRCC), as well as its response to therapy, are profoundly shaped by the tumor microenvironment (TME). Accumulating studies have consistently shown that, despite exhibiting extensive immune cell infiltration, ccRCC tumors frequently display impaired immune function or an immunosuppressive phenotype, highlighting the exceptional complexity of their immune landscape (Zhu et al., 2024; Qiu et al., 2025; Chen et al., 2024; Guijarro et al., 2025). These observations indicate that immune cell quantity alone does not adequately reflect effective antitumor immunity, and that the functional status of infiltrating immune cells is a critical determinant of immune competence. In line with this concept, transcriptomic profiling and comprehensive immune mapping studies have demonstrated that hypoxia, metabolic stress, aberrant angiogenesis, and stromal remodeling represent integral components of the ccRCC microenvironment, which together may influence both the functional states and spatial distribution of immune cells (Zhang et al., 2024; Liu et al., 2025; Zou et al., 2025). Collectively, these findings emphasize that immune activity in ccRCC is highly context-dependent and is strongly regulated by non-immune elements of the tumor niche, including endothelial and stromal compartments. Although VEGF/VEGFR-targeted therapies and immune checkpoint blockade (ICB) have improved patient outcomes over the past decade, the majority of patients eventually develop resistance, underscoring the urgent need to identify novel vulnerabilities and strategies to overcome therapeutic failure in ccRCC. Among potential targets, ionic homeostasis, particularly sodium ion flux (Zheng et al., 2023), has emerged as a critical regulator of cell fate; however, its mechanistic basis and clinical relevance in renal cancer remain insufficiently explored.

In parallel, dysregulation of regulated cell death pathways has emerged as an increasingly prominent theme in ccRCC research. A growing number of recent studies suggest that ferroptosis, necrosis-associated death programs, and stress-responsive cell death processes may contribute to both tumor biology and immune regulation in ccRCC (Guo et al., 2025; Kordowitzki and Grzeczka, 2025; Fu, 2025; Zou et al., 2024). Although the immunological consequences of distinct cell death modalities have not yet been fully delineated, accumulating evidence indicates that different death states can exert divergent effects on tumor immune activation, immune tolerance, and immune evasion (Chen et al., 2024; Zou et al., 2025). Nevertheless, each of these mechanisms displays essential limitations in the context of ccRCC. For example, VHL inactivation and subsequent HIF-dependent signaling pathways enhance apoptotic resistance in tumor cells (Chen et al., 2016; Kim et al., 2020). Ferroptosis sensitivity is heavily influenced by GPX4 activity and lipid metabolic plasticity, resulting in heterogeneous therapeutic responses (Forcina and Dixon, 2019; Tousignant et al., 2020; Hangauer et al., 2017). Moreover, in many renal cancer cell lines and clinical samples, expression of RIPK3 is frequently silenced through promoter methylation or other epigenetic mechanisms, thereby preventing activation of the necroptotic cascade (Geserick et al., 2015; Ganini et al., 2022). Consequently, ccRCC cell survival relies on a unique homeostatic landscape shaped by metabolic, ionic, and redox-linked signals, one that classical cell-death paradigms cannot sufficiently explain. Sodium overload–induced necrosis, proposed in recent years as a distinct ion-dependent form of regulated necrosis (Soll et al., 2024; Clemente et al., 2023), remains poorly characterized in cancer biology. TRPM4, a Ca^2+^-activated non-selective cation channel, is likely to serve as a central regulator of this sodium-driven death pathway; however, its functional role in tumors has mainly remained unclear.

In this study, we systematically elucidate the role of TRPM4 and sodium-overload–related cell death in ccRCC through integrated pan-cancer analyses, clinical datasets, and *in vitro* functional assays. We found that TRPM4 is markedly downregulated in ccRCC, in stark contrast to its elevated expression in most epithelial-derived malignancies. Functionally, restoring TRPM4 expression suppresses tumor cell proliferation, migration, and clonogenic capacity while inducing apoptosis, suggesting its potential role as a tumor suppressor in ccRCC. To more precisely quantify sodium-overload–associated cellular states, we developed the NECSO score. This scoring system not only predicts patient prognosis effectively but also delineates a transcriptional program enriched for hypoxia, inflammatory signaling, mitochondrial dysfunction, and disrupted ion homeostasis.

Despite the strength of the evidence presented, several limitations of this study should be acknowledged. First, the pan-cancer analyses, prognostic model construction, and evaluation of the immune microenvironment were primarily based on publicly available datasets, including TCGA, and therefore relied on a retrospective study design. As a result, these analyses may be subject to selection bias and incomplete clinical annotation. Although multiple statistical approaches and external validation cohorts were applied to mitigate these risks, prospective cohorts will ultimately be required to further validate the clinical applicability and robustness of the proposed model. Second, while *in vitro* functional assays clearly demonstrated a tumor-suppressive role for TRPM4 in clear cell renal cell carcinoma, *in vivo* validation was not included in the current study. Future investigations using appropriate mouse models will be necessary to confirm the contribution of TRPM4 and its associated gene network to tumor growth control, as well as to elucidate their impact on the tumor immune microenvironment under physiological conditions. Third, although a synergistic anti-tumor effect was observed between TRPM4 overexpression and NC1 treatment, the precise molecular mechanisms underlying this interaction remain incompletely defined. Further studies are warranted to clarify how TRPM4-mediated sodium influx converges with NC1-sensitive cell death pathways at both the signaling and effector levels. In summary, our findings identify TRPM4 as a key regulator of sodium homeostasis and highlight the NECSO score as a diagnostic- and prognosis-associated metric in ccRCC. Targeting dysregulated sodium-handling pathways may therefore represent a novel therapeutic strategy to attenuate renal cancer progression and overcome treatment resistance.

## Data Availability

The original contributions presented in the study are included in the article/Supplementary Material, further inquiries can be directed to the corresponding authors.
