# Correction: Development of a novel prognostic model based on TRPM4-Induced sodium overload–mediated cell death in kidney cancer

**DOI:** 10.3389/fcell.2026.1800735

**Published:** 2026-02-16

**Authors:** Wei Wang, Duo Zhao, Zijun Zhou, Bin Chen, Changwen Zhang, E. Du, Longchao Zhang

**Affiliations:** Department of Urology, Tianjin Institute of Urology, The Second Hospital of Tianjin Medical University, Tianjin, China

**Keywords:** bioinformatics, cell death, clear cell renal cell carcinoma (ccRCC), prognostic model, TRPM4, tumor immune microenvironment

**Affiliation** Department of Urology, Tianjin Institute of Urology, The Second Hospital of Tianjin Medical University, Tianjin, China was erroneously given as Second Hospital of Tianjin Medical University, Tianjin, China.

Authors Wei Wang, Duo Zhao, Zijun Zhou were erroneously omitted as equal contributing authors.

The original article has been updated.

