# Supplementary material for: Development of a novel prognostic model based on TRPM4-Induced sodium overload–mediated cell death in kidney cancer
Source: Front Cell Dev Biol. 2026 Jan 9;13:1755318. doi: 10.3389/fcell.2025.1755318 (PMC12827634; doi:10.3389/fcell.2025.1755318)
Supplement: Supplementary file 1 [file DataSheet1.docx]

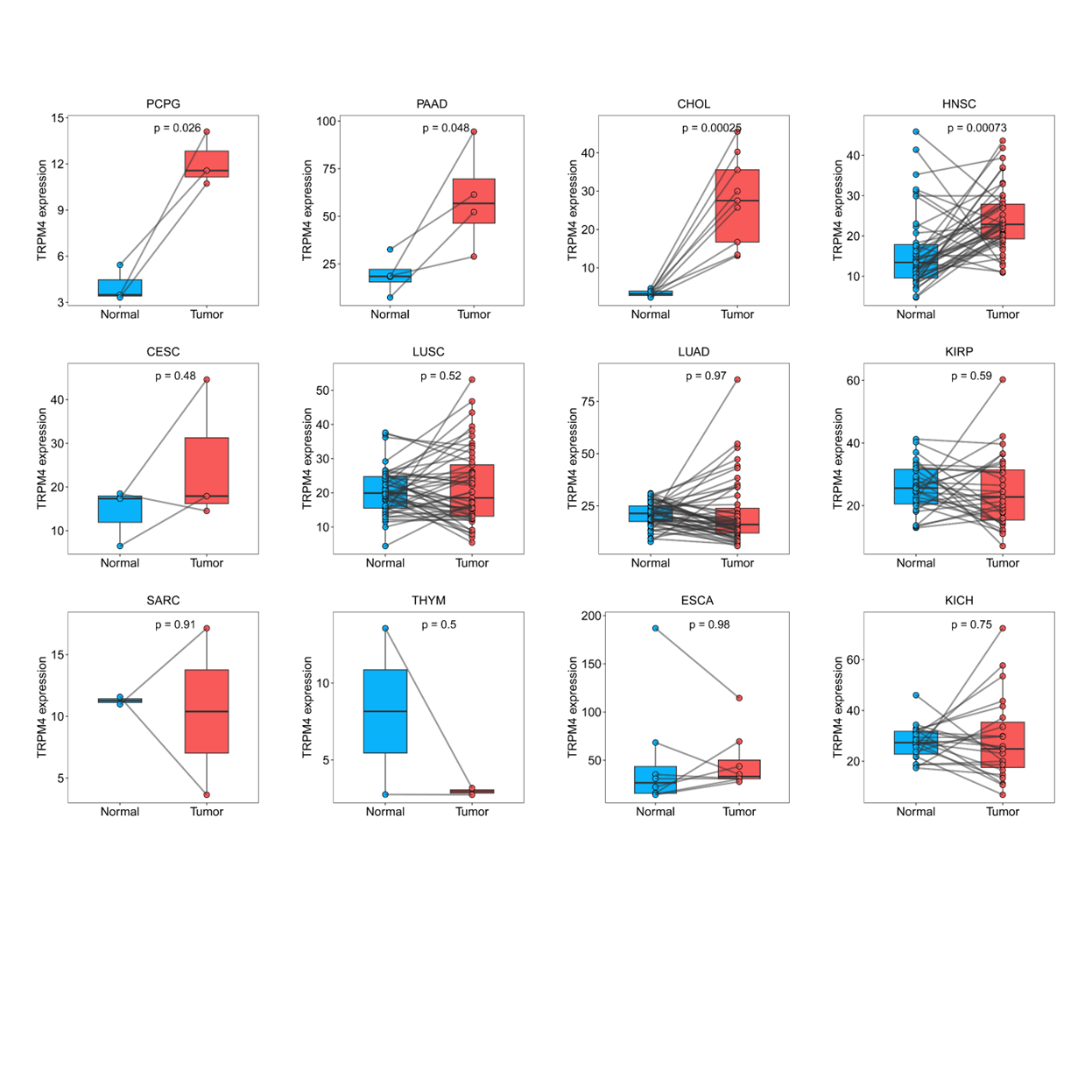


**Figure S1.** Comparison of TRPM4 expression between tumor and paired normal samples in 12 cancer types obtained from the TCGA database (* p < 0.05; ** p < 0.01; *** p < 0.001; ns, not significant).


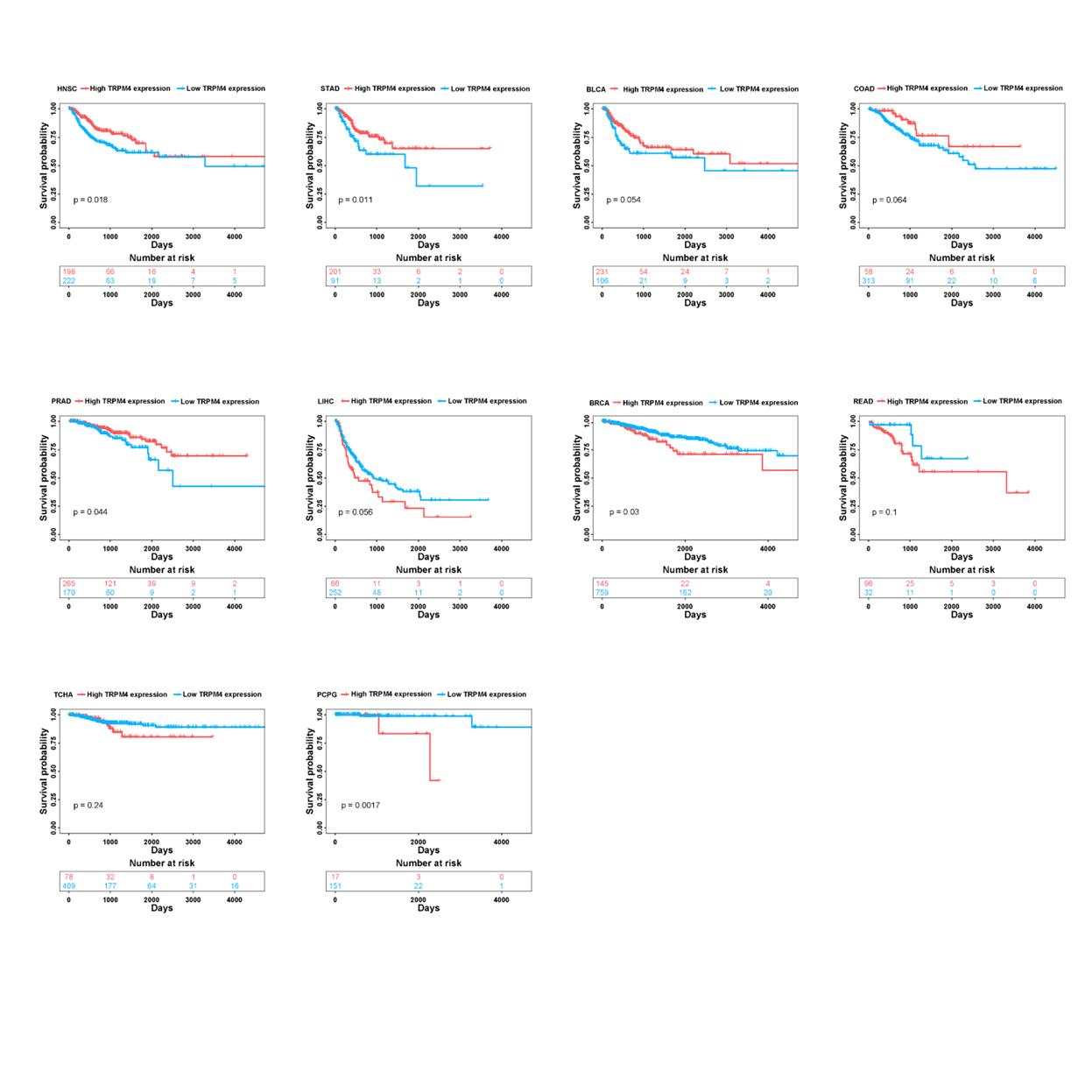


**Figure S2.** Kaplan–Meier analysis showing overall survival outcomes associated with high TRPM4 expression across pan-cancer cohorts.


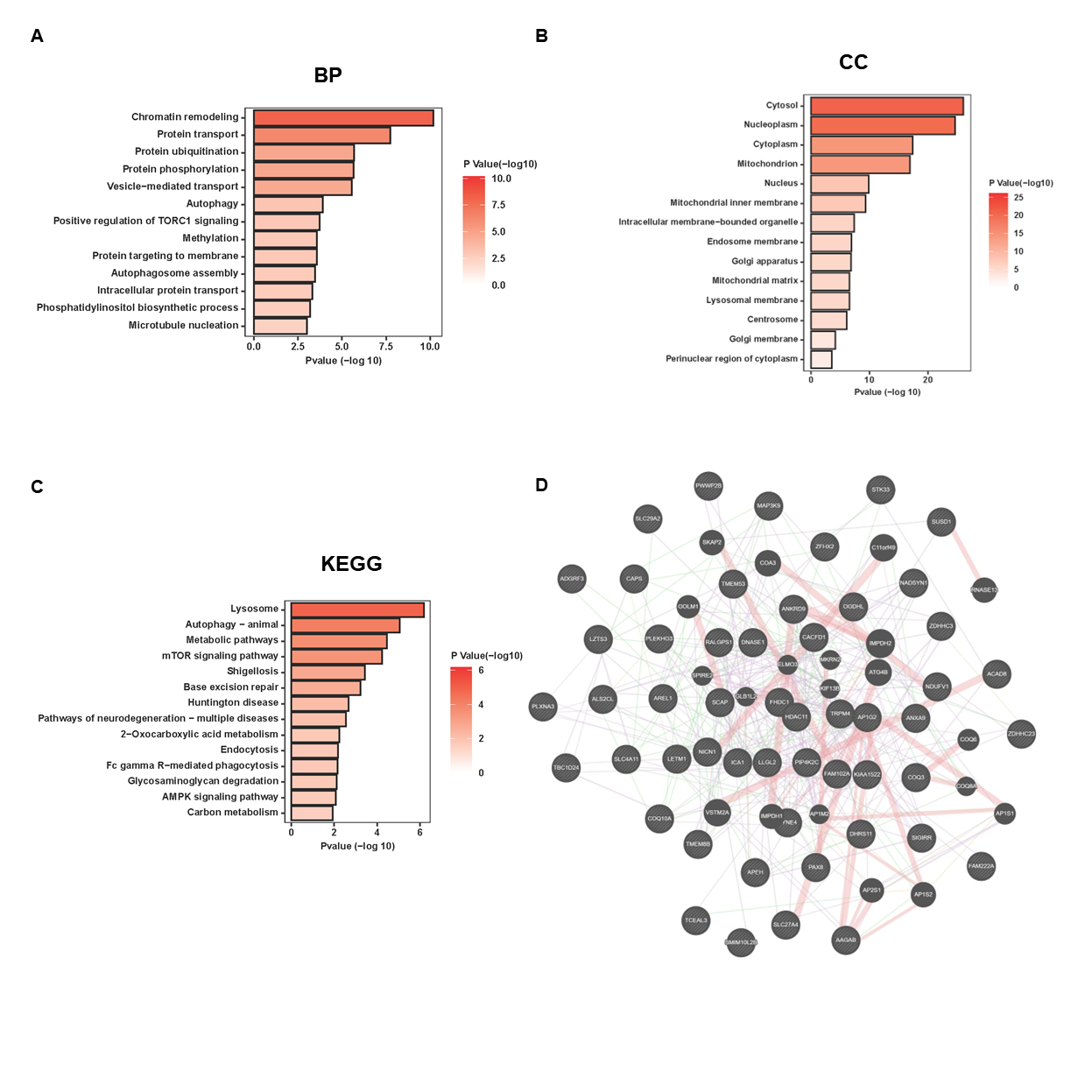


**Figure S3. GO analysis and PPI for DEGs.** GO analysis for DEGs in BP term(A), CC term(B), and KEGG term(C). (D) The PPI of 52 characterized genes.


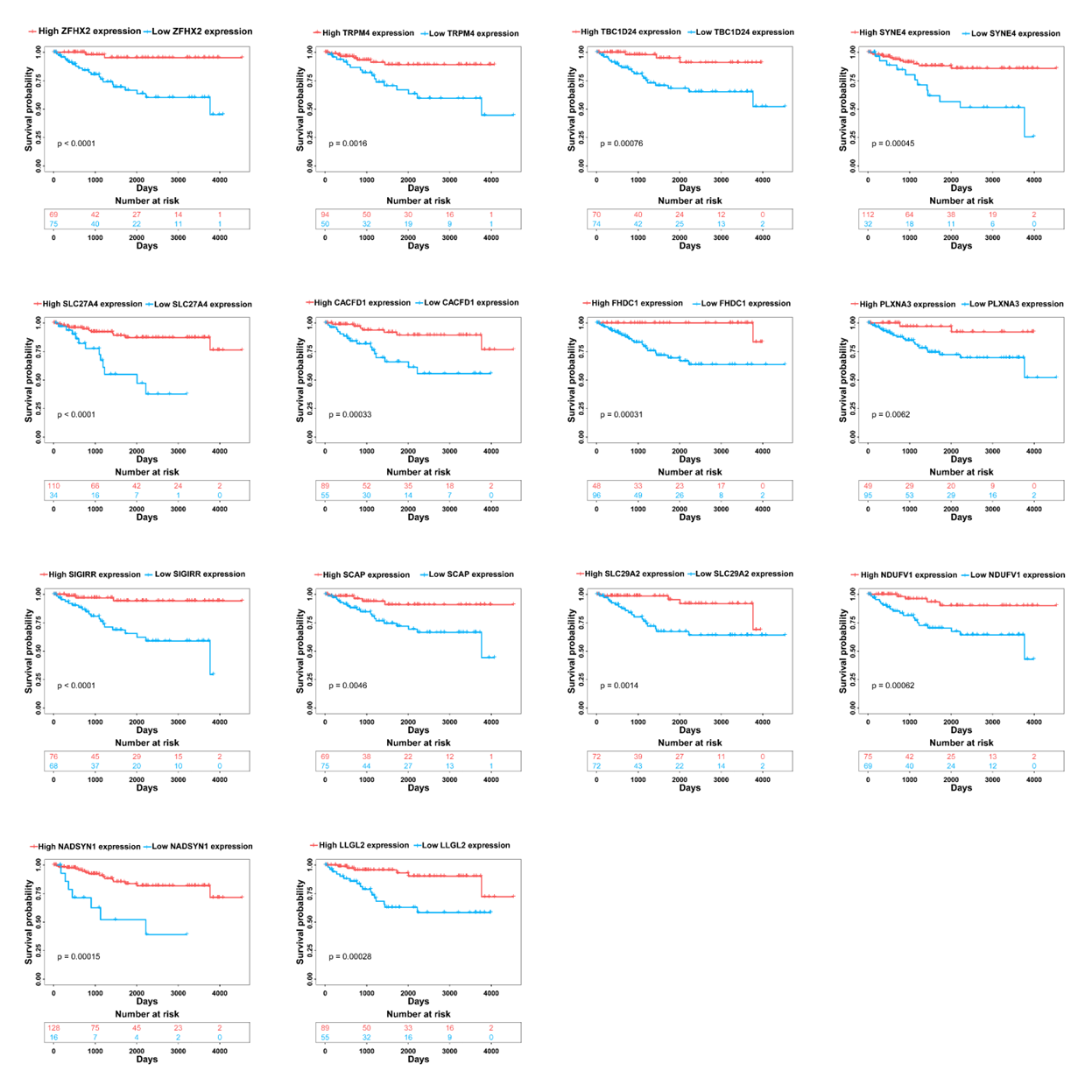


**Figure S4.** The Kaplan-Meier curve shows the prognostic differences between high and low expression of the 14 gene diagnostic features.


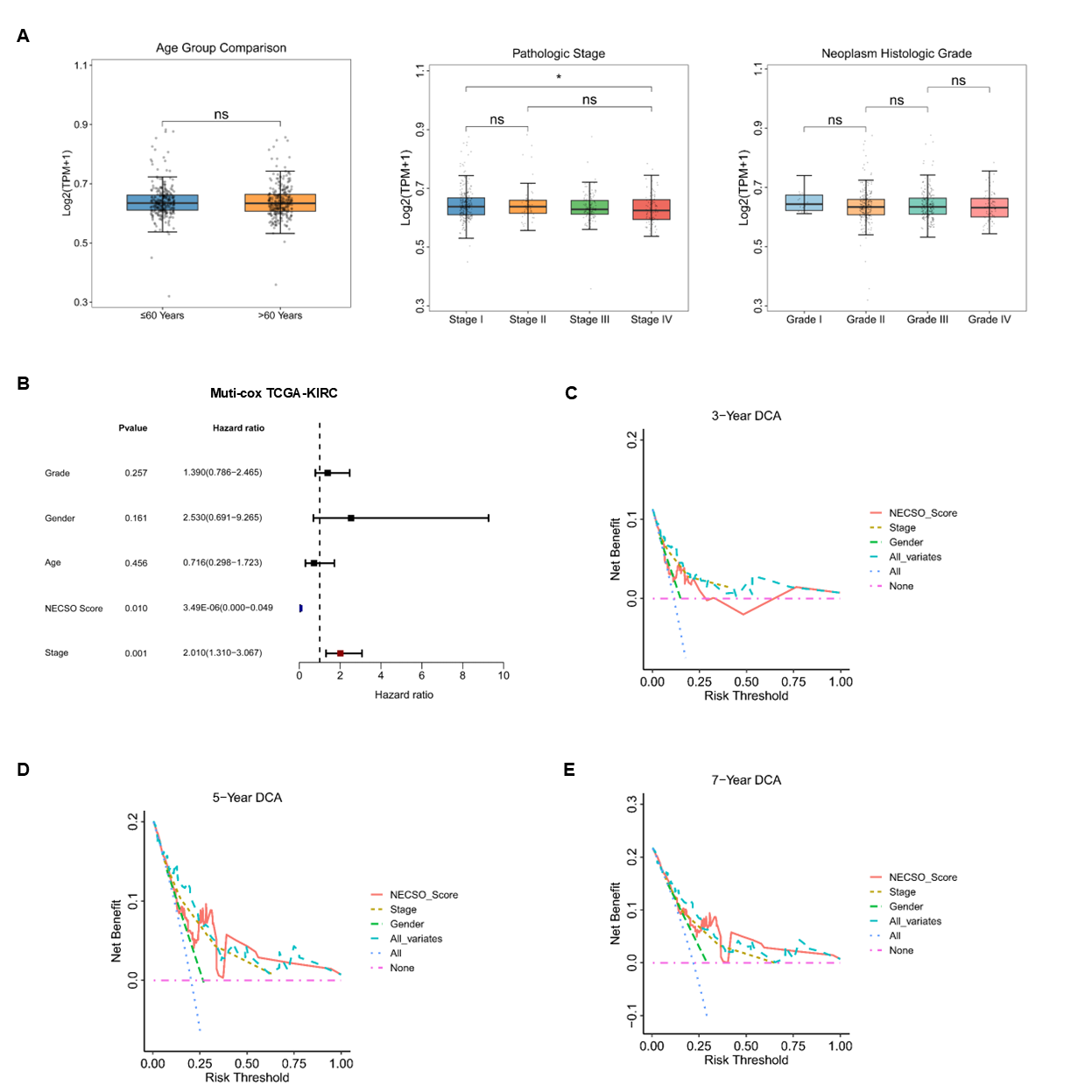


**Figure S5.** **NECSO Score Correlates with Clinical-Pathological Features and Prognosis in TCGA-KIRC** (A) Distribution of NECSO scores across age, pathological stage, and pathological grade subgroups. (B) Multivariate Cox proportional hazards analysis incorporating NECSO Score and clinical parameters. (C-E) Decision curve analysis of the predictive model at 3, 5, and 7 years in the TCGA-KIRC cohort.


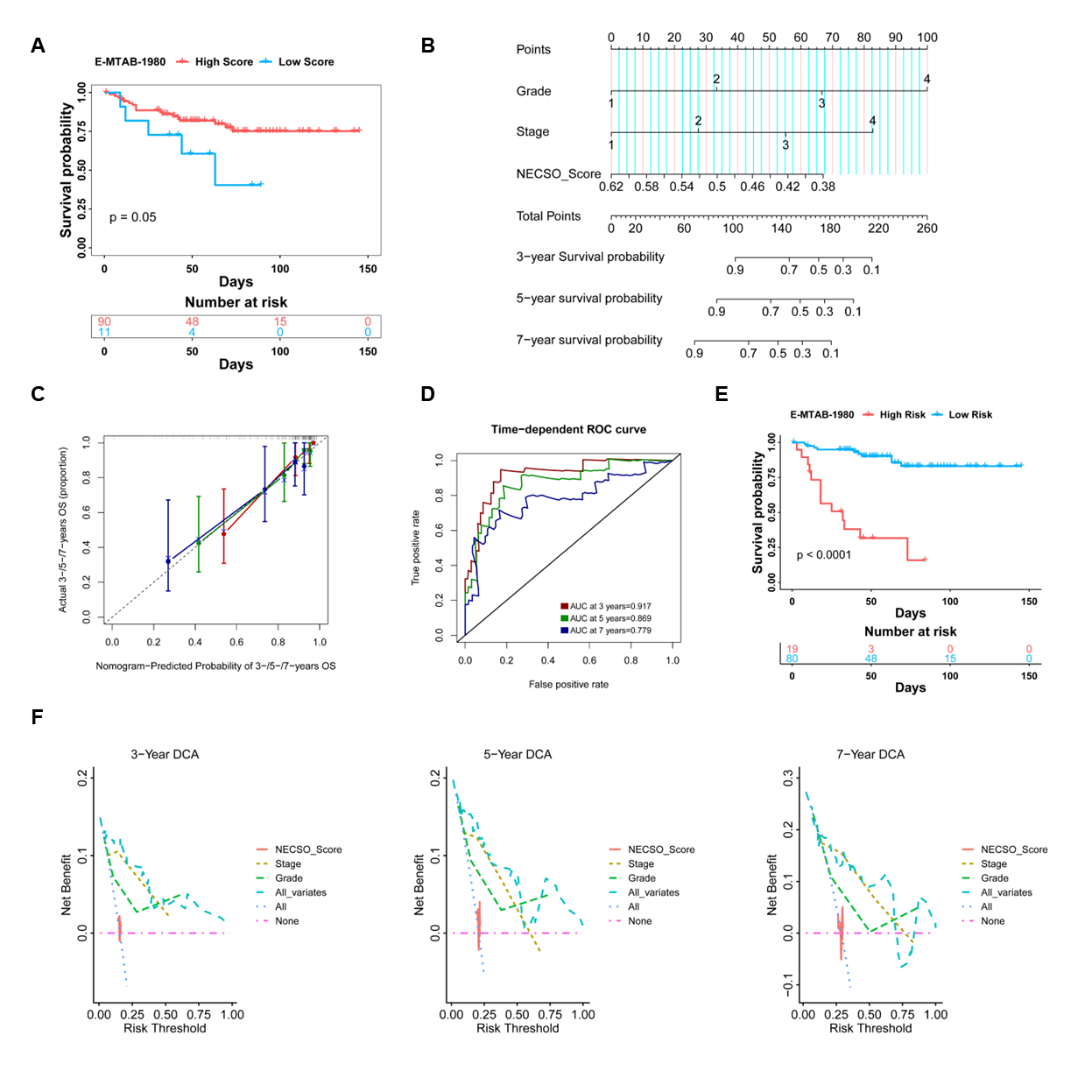


**Figure S6.** **NECSO Score Correlates with Clinical-Pathological Features and Prognosis in E-MTAB-1980** (A) Kaplan-Meier survival analysis revealed that patients in the High NECSO Score group had significantly better OS than those in the Low Score group. (B) Nomogram predicting 3-, 5-, and 7-year survival probabilities for patients in the E-MTAB-1980 cohort. (C) Calibration plots showing the consistency between nomogram-predicted and actual survival probabilities at 3, 5, and 7 years. (D) Time-dependent ROC analysis of the combined model. (E) Kaplan-Meier curves comparing survival between nomogram-defined High- and Low-risk groups. (F) Decision curve analysis of the predictive model at 3, 5, and 7 years in the E-MTAB-1980 cohort.


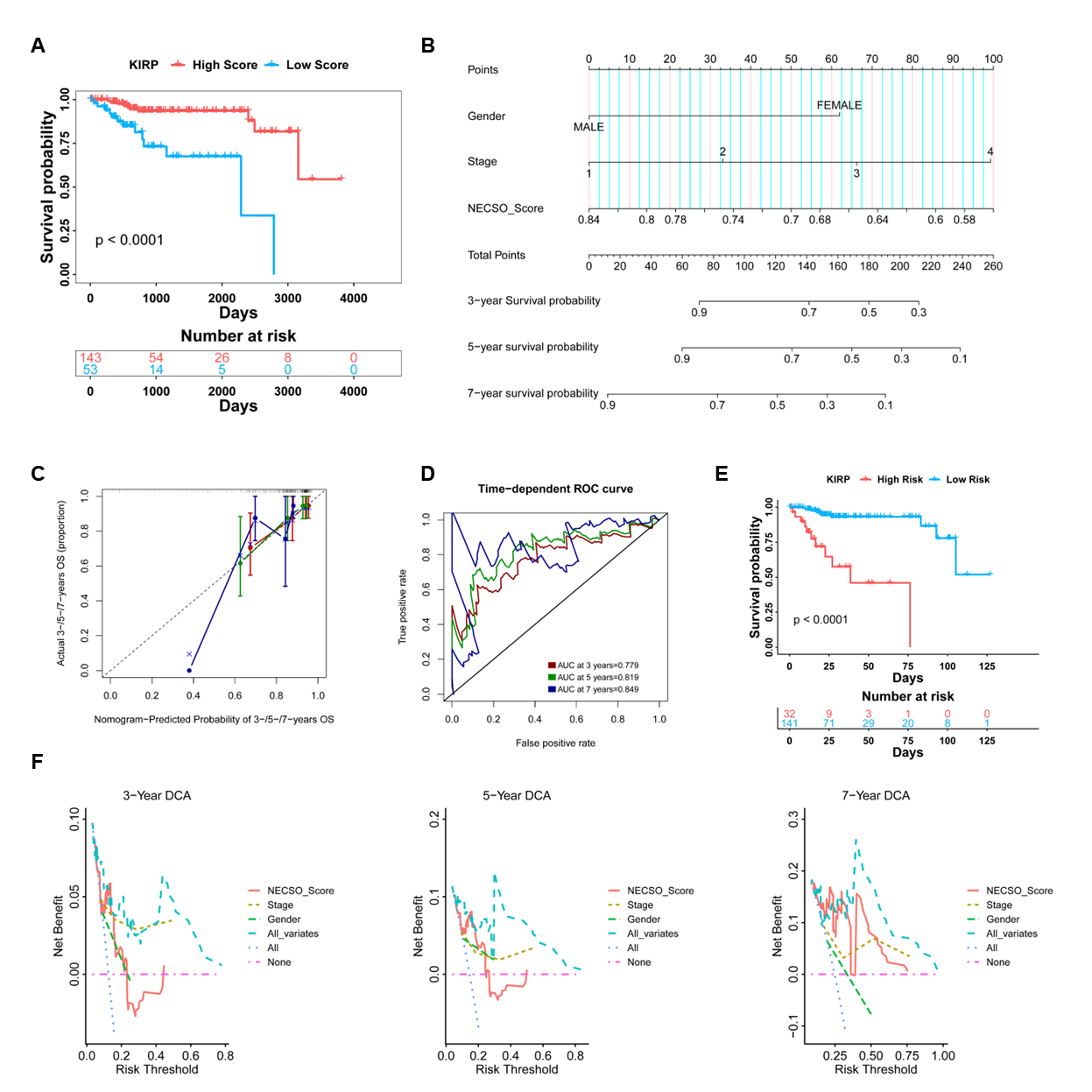


**Figure S7.** **NECSO Score Correlates with Clinical-Pathological Features and Prognosis in TCGA-KIRP** (A) Kaplan-Meier survival analysis revealed that patients in the High NECSO Score group had significantly better OS than those in the Low Score group. (B) Nomogram predicting 3-, 5-, and 7-year survival probabilities for patients in the TCGA-KIRP cohort. (C) Calibration plots showing the consistency between nomogram-predicted and actual survival probabilities at 3, 5, and 7 years. (D) Time-dependent ROC analysis of the combined model. (E) Kaplan-Meier curves comparing survival between nomogram-defined High- and Low-risk groups. (F) Decision curve analysis of the predictive model at 3, 5, and 7 years in the TCGA-KIRP cohort.


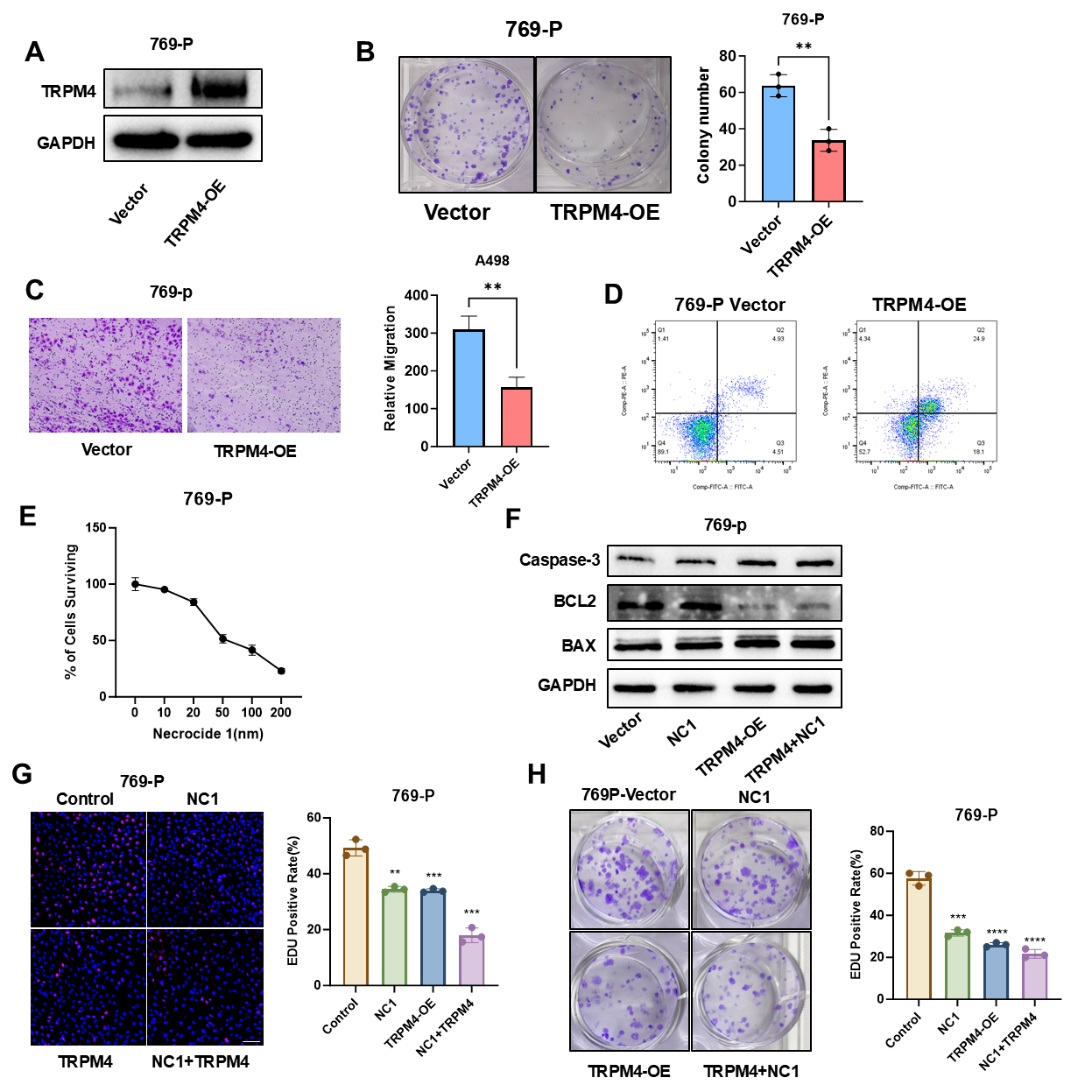


**Figure S8. Overexpression of TRPM4 inhibits RCC progression while promoting programmed cell death in 769-P cells.** (A) Detection of TRPM4 expression in 769-P cells by Western blot analysis. (B) Colony formation comparison between TRPM4-OE and control cells. (C) Transwell assays were used to evaluate the migration of TRPM4-OE cells compared to controls. (D) Flow cytometry analysis of apoptosis levels in control and TRPM4-OE cells. (E) Percentage of viable A498 cells following exposure to varying concentrations of NC1. (F) Detection of Caspase-3, Bax, and BCL-2 expression in A498 cells by Western blot analysis. (G) EdU assay showing that TRPM4 overexpression and the NC1 inhibitor suppress A498 cell proliferation. Scale bar = 200 µm. (H) Colony formation comparison between control cells, NC1, TRPM4-OE, and NC1+TRPM4-OE.
